# Effects of Wet and Dry Micronization on the GC-MS Identification of the Phenolic Compounds and Antioxidant Properties of Freeze-Dried Spinach Leaves and Stems

**DOI:** 10.3390/molecules27238174

**Published:** 2022-11-24

**Authors:** Renata Różyło, Jolanta Piekut, Dariusz Dziki, Marzena Smolewska, Sławomir Gawłowski, Agnieszka Wójtowicz, Urszula Gawlik-Dziki

**Affiliations:** 1 Department of Food Engineering and Machines, University of Life Sciences in Lublin, 28 Głęboka Str., 20-612 Lublin, Poland; 2 Department of Agricultural, Food and Forestry Engineering, Bialystok University of Technology, 45E Wiejska Str., 15-351 Białystok, Poland; 3 Department of Thermal Technology and Food Process Engineering, University of Life Sciences in Lublin, 31 Głęboka Str., 20-612 Lublin, Poland; 4 Faculty Chemical Laboratory, Bialystok University of Technology, 45E Wiejska Str., 15-351 Białystok, Poland; 5 Department of Biochemistry and Food Chemistry, University of Life Sciences in Lublin, 8 Skromna Str., 20-704 Lublin, Poland

**Keywords:** spinach leaves, spinach stems, spinach by-product, micronization, grinding, freeze drying, particle size, phenolic compounds, antioxidant activity

## Abstract

Micronization is an emerging technology used in food production, in which the size of particles is reduced to microns in the processing of plant raw materials and by-products, thus making it an interesting research topic. Spinach stems are by-products of spinach leaf processing, but there is little information regarding their processing and possible reuse. In this study, wet and dry ball mill micronization, in combination with freeze drying, was used to process spinach stems and leaves to obtain functional powders. The color and particle size of the micronized spinach leaf and stem powders were evaluated. The antioxidant activity (AA) of the powders and phenolic compounds present in them were determined using GC-MS analysis. The results obtained showed that the dry micronization of leaves and stems resulted in smoother and brighter powders than wet micronization. Significantly smaller particle sizes were achieved using the dry micronization of the leaves and stems (Dv50 = 19.5 and 10.1 µm, respectively) rather than wet micronization (Dv50 = 84.6 and 112.5 µm, respectively). More phenolic compounds, such as o-coumaric acid and gallic acid, were extracted from the dry-micronized powders. The dry micronization of the stems significantly increased the total phenolic content, and the AA of these powders was also increased. These findings demonstrate that spinach leaves and stems subjected to dry micronization can be valuable functional components of food.

## 1. Introduction

Micronization is an intensive grinding operation used to reduce the particle size of food materials to microns. Conventional micronization techniques are used in the pharmaceutical industry, but in recent decades, numerous studies have reported their great potential in the food industry [1]. Therefore, research on micronized plant materials has gained increasing interest. Studies carried out thus far indicate the positive effects of the significant reduction in the particle size of plant materials on their functional and physicochemical properties [2]. Such changes in various plant materials, including by-products, are not yet fully understood. Therefore, research on this subject is necessary.

Scientific evidence shows that diets rich in plant products protect humans against lifestyle diseases, such as cancer, obesity, and cardiovascular disease. In particular, green leafy vegetables are considered to have significant health-promoting effects [3,4]. They can be used in combination or as single food ingredients, especially in functional and nutraceutical applications. Diets in which green leafy vegetables are abundant have the potential to delay the onset of age-related diseases [3].

Spinach (*Spinacia oleracea* L.) is widely considered as a functional food due to its diverse nutritional composition, including vitamins, minerals, phytochemicals, and bioactive ingredients that promote health [4,5].

Spinach leaves are rich in minerals, including valuable minerals such as calcium, magnesium, potassium, and iron [6,7]. Recent research has suggested that spinach is a source of fatty acids, amino acids, organic acids, and vitamins, and that spinach contains palmitic (C16:0), elaidic (C18:1cis), linoleic (C18:2n-6), and linolenic (C18:3n-3) fatty acids. Spinach leaves have a total fatty acid content of 18.67 mg/100 g based on their dry weight, and their saturated and unsaturated fatty acid contents are 16.01% and 83.88%, respectively. A total of 118 compounds representing organic acids, phenolic acids, and flavonoids have been identified in spinach extracts [3]. In addition, spinach is low in calories and rich in proteins and fiber [6].

In addition to fresh spinach available on the market, the demand for spinach powder is increasing [8,9]. Spinach powder is a rich source of proteins, fiber, antioxidants, and minerals, rendering it a potential ingredient for functional food production [9]. The results of the research conducted on dehydrated spinach thus far have reported that spinach powder has 8.2% crude fiber, 19.2% protein, 1304 mg/100 g calcium, and 40.4 mg/100 g iron [10].

Spinach powder is usually prepared by the drying of spinach leaves or spinach juice. Thus far, various methods of spinach drying have been tested, such as convection drying [11], freeze drying [12], spray drying [13], microwave drying [14], and high-electric-field drying [15].

Some of the drying methods, such as hot air drying, sun drying, vacuum freeze drying, and spray drying, have been shown to reduce the total contents of phenols, flavonoids, chlorophyll, and ascorbic acid, and the antioxidant activity (AA). Spinach powders prepared using spray drying had the highest AA, whereas those prepared using hot air drying had the lowest AA [16]. The drying process, regardless of the method, leads to color changes [11,17,18,19]. Both convection drying with hot air (40, 50, 60 °C) and microwave drying (180, 360, and 600 W) resulted in significant changes in the color parameters, where the L* and a* values of the samples increased, whereas their b* values decreased [20]. Some studies have also proved that the degradation of the color and L-ascorbic acid occurs during hot air drying [18]. The drying temperature influences the levels of lutein, β-carotene, and chlorophyll in spinach leaf tissues [21]. As reported in previous studies, freeze drying resulted in a lower degradation of chlorophyll than hot air drying. Moreover, the samples dried using freeze drying had higher concentrations of phenolics, flavonoids, vitamins, and glucosinolates [12].

Based on the abovementioned findings, it is reasonable to use freeze drying to process spinach leaves. In addition to the leaves, the spinach stems, which are by-products, can be used as functional additives. Previous studies have shown that both the leaves and stems of spinach contain significant amounts of Fe, Mn, and Zn. The stems have a higher content of Na and K than the leaves, whereas the contents of other elements are higher in the leaves [22]. Thus far, no studies have compared the antioxidant properties of freeze-dried leaves and stems. Moreover, the micronization of such products has not been tested. Micronization is an emerging technology, and research on micronization in food production is scarce [1,22].

Therefore, this study aimed to determine the effects of wet and dry ball mill micronization on the properties of freeze-dried spinach leaves and stems. The color, particle size, phenolic compounds, and AA of the micronized powders from spinach leaves and stems were analyzed.

## 2. Results and Discussion

### 2.1. The Appearance and Color Parameters of Powders from Micronized Spinach Leaves and Stems

**Figure 1:**
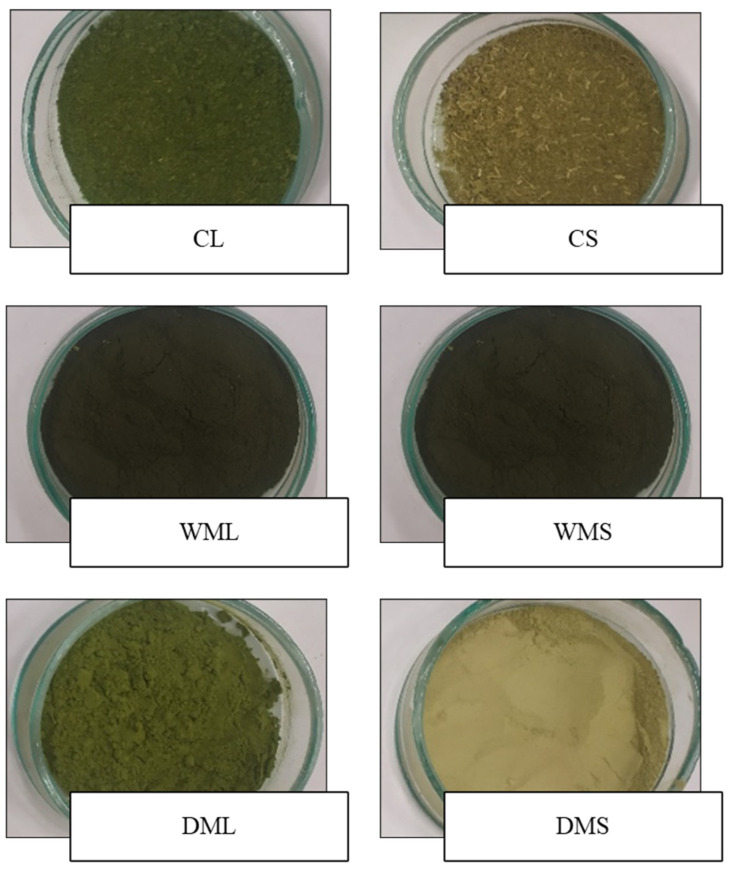
Appearance of micronized spinach leaves and stems. CL-control freeze-dried spinach leaf powder without micronization, CS-control freeze-dried spinach stem powder without micronization, WML—wet-micronized freeze-dried spinach leaf powder, WMS-wet-micronized freeze-dried spinach stem powder, DML-dry-micronized freeze-dried spinach leaf powder, DMS-dry-micronized freeze-dried spinach stem powder.

Images showing the appearance of the spinach leaf and stem powders are presented in Figure 1. The appearance of the powders was dependent on the methods used, such as freeze drying without the micronization of the spinach leaves (CL) and stems (CS); wet micronization plus freeze drying of the spinach leaves (WML) and stems (WMS); and freeze drying plus dry micronization of the spinach leaves (DML) and stems (DMS). In addition, dry micronization resulted in better products, i.e., finer and smoother powders from spinach leaves and stems, than wet micronization. Furthermore, the powders from the DML were characterized by a more intense green color than those from the DMS.

**Figure 2:**
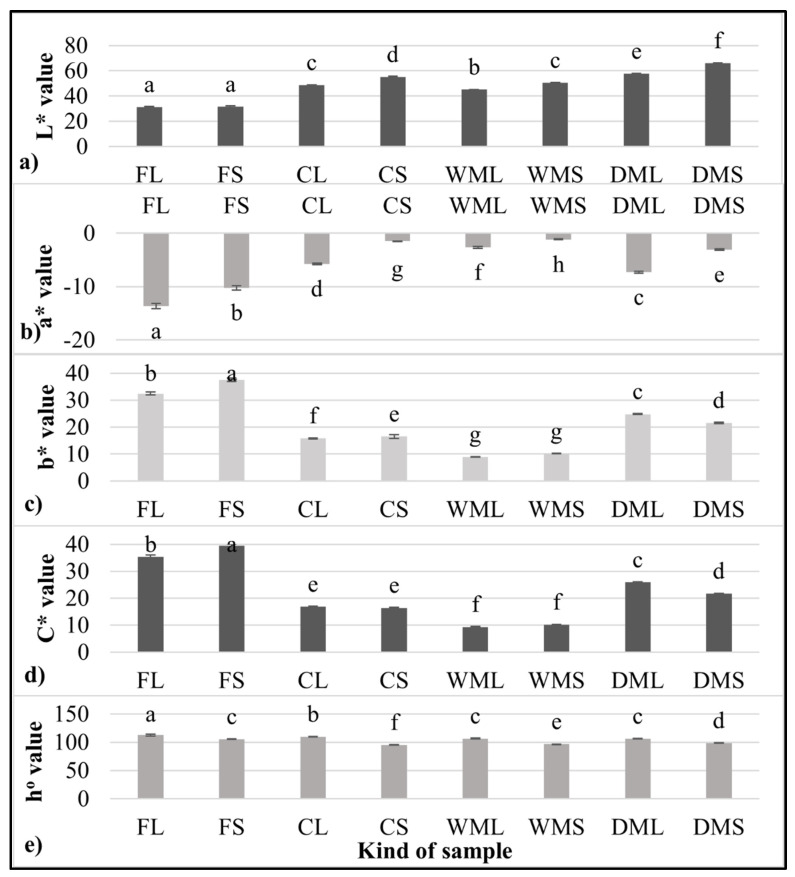
Color parameters of fresh and micronized spinach leaves and stems. (**a**) L* value; (**b**) a* value; (**c**) b* value, (**d**) C* value; (**e**) h^o^ value FL-fresh spinach leaves, FS-fresh spinach stems, CL-control freeze-dried spinach leaf powder without micronization, CS-control freeze-dried spinach stem powder without micronization, WML-wet-micronized freeze-dried spinach leaf powder, WMS-wet-micronized freeze-dried spinach stem powder, DML-dry-micronized freeze-dried spinach leaf powder, DMS-dry-micronized freeze-dried spinach stem powder, a–h-values in the same column marked with different letters are significantly (*α* = 0.05) different.

In general, the intensity of the green color is higher in leaf powders than in stem powders. This was confirmed by measuring the color values of the leaves and stems (Figure 2a–e), which, despite insignificant differences in the L* brightness parameter, differed significantly in the a* and b* parameters. The significantly lower value of a* (−13.7) determined for the fresh leaves of the spinach (FL) compared with the fresh stems (FS) (−10.2) indicated a significantly higher share of green color in the leaves. This study aimed to use production waste, i.e., spinach stems, which, despite their low-intensity green color, can act as an important source of other ingredients. Therefore, the spinach stems were subjected to further analysis.

The samples obtained by wet micronization (WML and WMS) showed a dark green color, suggesting that the partial degradation of the chlorophyll may have already occurred. Chlorophyll degradation can be attributable to temperature changes during wet micronization. To prevent browning, the temperature during wet micronization was monitored, and the wet micronization process was ended at 30 °C, but the color of the product eventually darkened. The results of other studies [23] on the storage of green vegetables at different temperatures also suggest that the rates of chlorophyll degradation and, thus, color degradation increase with an increasing storage temperature. In addition to chlorophyll degradation, enzymatic browning can take place due to the activity of polyphenol oxidase, which is faster under normal atmospheric pressure [22]. In the present study, the samples (DML and DMS) that were first freeze-dried under a reduced pressure were green in color.

### 2.2. Characteristics of the Particle Sizes of the Micronized Spinach Leaf and Stem Powders

The particle sizes of the spinach leaf and stem powders are presented in Table 1. As expected, both wet (WML and WMS) and dry micronization (DML and DMS) resulted in changes in the particle size of the spinach leaf and stem powders. The mean particle size of the spinach leaf powders measured on the surface area D [3;2] decreased from 65.6 (CL) to 27.5 µm (WML) after wet micronization and to 6.4 µm after dry micronization. The particle size of the spinach stem powders was significantly higher than that of the leaf powders, and wet and dry micronization decreased the mean particle size from 74.5 (CS) to 40.0 µm (WMS) and 3.3 µm (DMS), respectively. The Dv50 of 50% of the particles is the most commonly used particle size discriminant. The control samples of the spinach leaves without micronization had 50% of the particles below 182.3 µm (CL); however, this value decreased to 84.6 µm (WML) and 19.5 µm (DML) after wet and dry micronization, respectively. The control samples of the spinach stem powders had 50% of the particles below 210.7 µm (CS); however, this value decreased significantly to 112.5 µm (WMS) and 10.1 µm (DMS) after wet and dry micronization, respectively.

Similar trends were observed for the Dv10 and Dv90 indicators. The spinach leaf and stem controls had a Dv90 index of 666.3 (CL) and 743.7 µm (CS), respectively. These values decreased to 252.7 µm (WML) and 353.0 µm (WMS) and to 62.1 (DML) and 40.8 µm (DMS), respectively, after wet and dry micronization. Micronization is an emerging technology that is used more often in the pharmaceutical industry [24]. Research on the application of this technology to plant products is scarce. Previous studies confirm that micronization is a high-shear mechanical operation that can reduce the particle size of food materials to microns [1].

### 2.3. GC-MS Identification of Phenolic Compounds in the Micronized Spinach Leaf and Stem Powders

**Figure 3:**
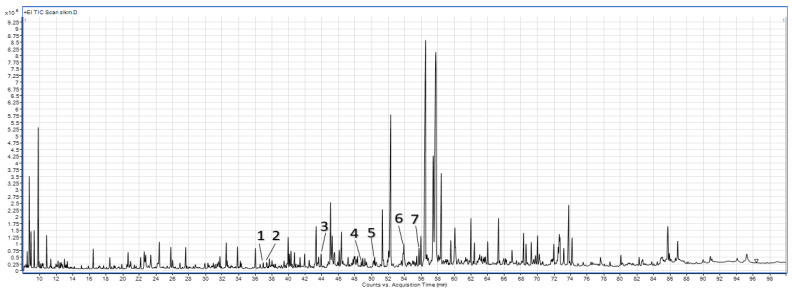
Sample chromatogram of phenolic compounds from the GC-MS analysis of the wet-micronized freeze-dried spinach leaf powders. 1—3-Hydroxyphenylacetic acid, 2—4-hydroxyphenylacetic acid, 3—o-coumaric acid, 4—*p*-coumaric acid, 5—gallic acid, 6—ferulic acid, 7—caffeic acid.

The following phenolic compounds were identified from the spinach leaf (CL, WML, and DML) and stem (CS, WMS, and DMS) powders (Table 2, Figure 3): 3-hydroxyphenylacetic acid, 4-hydroxyphenylacetic acid, o-coumaric acid, *p*-coumaric acid, gallic acid, ferulic acid, and caffeic acid. Significant differences were observed in the control samples of the spinach leaf and stem powders. The leaf powders (CL) showed higher contents of 3-hydroxyphenylacetic acid, gallic acid, ferulic acid, and caffeic acid than the stem powders (CS). However, the stem powders (CS) showed significantly higher contents of 4-hydroxyphenylacetic acid, o-coumaric acid, and *p*-coumaric acid than the leaf powders (CL). Both DML and DMS contributed to an increase in the contents of o-coumaric acid and gallic acid. In addition, significantly higher contents of 3-hydroxyphenylacetic acid, 4-hydroxyphenylacetic acid, and *p*-coumaric acid were observed in the leaves after dry micronization. WML and WMS significantly increased the contents of 3-hydroxyphenylacetic acid, 4-hydroxyphenylacetic acid, and gallic acid. However, wet micronization reduced the contents of *p*-coumaric acid and caffeic acid. Previous studies on the micronization of grape pomace and fiber concentrate have shown that micronization increases the extraction of phenolic compounds, especially catechin and epicatechin [25].

### 2.4. Total Phenolic Content (TPC) and AA of the Micronized Spinach Leaf and Stem Powders

As reported in [26], spinach is a rich source of phenolic compounds and contains primarily patuletin, spinacetin, and glucuronide derivatives, which are responsible for its antioxidant properties (AA). The TPC and AA of the spinach leaf and stem powders are presented in Table 3. The lowest TPC—0.3 mg GAE (gallic acid equivalent)/g DM—was observed in the wet-micronized and freeze-dried stem powder (WMS), whereas the dry-micronized spinach stem powder (DMS) showed an approximately twofold higher TPC (0.67 mg GAE/g DM).

Wet micronization before freeze drying had no significant influence on the TPC in both the leaves and stems. Interestingly, the method of micronization had a significant influence on the AA. The highest activity (the lowest value of EC50) was observed in the extracts obtained from the dry-micronized and freeze-dried stem powders and control leaves. This tendency was observed in both the DPPH and ABTS. The wet micronization of the stems (WMS) resulted in the lowest AA. In the leaves, wet micronization (WML) also decreased the AA compared with the controls. Moreover, a strong and positive correlation was observed between the ABTS and DPPH (*r* = 0.941, *p* < 0.05). Previous studies have demonstrated remarkable changes to the food matrix introduced by micronization techniques. Reducing the particle size of biological materials to microns improves their functional properties and antioxidant capacity [1]. Studies on the micronization of grape pomace and fiber concentrate have shown that micronization increases the antioxidant capacity, evaluated using ABTS and ORAC tests [25]. In addition, some studies have shown that the micronization of olive pomace leads to a redistribution of the fiber fraction (from insoluble to soluble), a reduction in the lignin content, and a change in the granule morphology. In this way, micronization transforms the raw materials into new powders with improved technological and functional properties [27]. Similarly, in the present study, the dry micronization of freeze-dried spinach leaves (DML) and stems (DMS) had positive effects on the TPC and AA, and wet micronization (WML and WMS) resulted in unfavorable changes, as indicated by a decrease in the AA and, as mentioned earlier, in the darkening of the color of the spinach pulp. The correlation analysis showed a positive relationship between the L* lightness parameter and the TPC (*r* = 0.861, *p* < 0.05).

## 3. Materials and Methods

### 3.1. Materials

The raw material used in this study was New Zealand spinach (*Tetragonia tetragonioides*) grown from selected seeds (Torseed, Toruń, Poland) on the experimental plot in the Lublin region. The fresh spinach sprigs were divided into leaves and stems and were stored in a refrigerator at 2–4 °C for 24 h before processing.

### 3.2. Micronization of the Spinach Leaves and Stems

The control samples obtained from the leaves (CL) and stems (CS) were pulped using a knife grinder (60 s) and then freeze-dried using an ALPHA 1–4 lyophilizer (30 °C, 63 Pa), as described in previous studies [28]. The micronization of the fresh (wet micronization) or freeze-dried (dry micronization) samples was carried out using a ball mill (Pulverisette 6, Fritsh. Germany). The WML and WMS samples were pulped (60 s, knife grinder) in the same way as the control samples and then micronized (300 rpm, ball mill) for 15 min, so as to avoid exceeding the temperature of 30 °C (significant browning of the pulp was observed at higher temperatures). After wet micronization, the pulp was freeze-dried. The DML and DMS samples were pulped using a knife grinder (60 s), as mentioned above, and then freeze-dried. After lyophilization, the dry samples were micronized with the same parameters (300 rpm) as those for the wet micronization.

### 3.3. Color Measurements

Color measurements of the analyzed samples were carried out on the CIE L* a* b* scale using a colorimeter (4 Wave CR30–16, Planeta, Tychy, Poland) [28,29]. On this scale, the parameter L* (ranging from 0 to 100) denoted the brightness of the material. With respect to the color index a* (ranging from −150 to +100), negative values indicated the share of green color, and positive values indicated red. However, for the b * index (ranging from −100 to +150), negative values indicated the blue color, and positive values indicated yellow. The parameter C* (chroma) represented the color intensity, and h^o^ represented the color angle.

### 3.4. Particle Size Analysis

Analyses of the particle size of the control (CL and CS) and micronized (WML, WMS, DML, and DMS) spinach leaves and stems were carried out using a Mastersizer 3000 (Malvern Instruments Ltd., Malvern, UK) [30]. The measurements were performed using the dry dispersion method (Aero S). The obtained results were expressed as follows: the volume-weighted average particle dimensions (D [4;3] (µm)) and average dimensions weighted by the surface area D [3;2] (µm). The Dv50, Dv10, and Dv90 particle sizes in microns were also determined, according to which 50%, 10%, and 90% of the samples were smaller than the values determined by these parameters, respectively.

### 3.5. Extraction and Derivatization of the Phenolic Compounds

From the samples of the powdered raw materials, 5 g was extracted thrice with 40 mL of 80% acidified methanol (acidified with hydrochloric acid to pH = 2) at 40 °C by sonication. The supernatant was evaporated under reduced pressure to remove the methanol.

The phenolic compounds were extracted using 2 × 10 mL portions of diethyl ether/ethyl acetate (*v*/*v* 1:1). The collected eluent was dried over anhydrous sodium sulfate and then evaporated to dryness using a rotary evaporator under reduced pressure. The extracted dry residue was derivatized with 100 µL of N,O-bis(trimethylsilyl) trifluoroacetamide, together with 1% trimethylchlorosilane (for the derivatization of GC, Supelco, Bellefonte, PA, USA) and 200 µL of pyridine (anhydrous, 99.8%, Sigma-Aldrich, St. Louis, MO, USA). The contents were heated at 60 °C for 1 h.

### 3.6. Separation and Detection of Phenolic Compounds by GC-MS Method

The separation and detection of the phenolic compounds were carried out following the method of Różyło et al. [31] using a 7890B GC System with a 7000C GC/MS Triple Quad Mass Detector (Agilent Technologies, Santa Clara, CA, USA). The standards of 25 different phenolic acids and 8 compounds from the flavonoid group were analyzed. All the standards were purchased from Sigma-Aldrich. Of these, 7 phenolic acids were quantified in the extracts. An HP-5 ms capillary column with fused silica (30 m × 0.25 mm × 0.25 µm, Agilent Technologies) was used in the separation process. The injection (the volume of the sample injected was 1 µL) temperature was maintained at 260 °C, and the carrier gas flow rate was maintained at 1 mL·min^−1^ (helium). The chromatographic analysis was conducted based on a validated procedure [32]. Temperatures from 40 to 300 °C were programmed at a rate of 3 °C min^−1^ (1:10 split) to separate the compounds. The process detection was performed in the full scan mode from 45 to 600 m/z.

### 3.7. Preparation of the Extracts for Further Research on the Phenols and Antioxidants

The powders were extracted from the spinach leaves and stems (1 g) for 30 min in 5 mL of a methanol–water mixture (1:1, *v*/*v*). Then, the extracts were centrifuged for 15 min, the residue was re-extracted with the same amount of methanol (5 mL), and the combined extracts were stored (−20 °C, dark).

### 3.8. Assessment of the Total Phenolics and AA

The TPC was determined using the Folin–Ciocalteu method [33] and was expressed in mg as GAE. The AA was assessed using DPPH radical scavenging methods [34] and ABTS [35]. A plate spectrophotometer (BioTek, Model Epoch2TC, S/N 15120115) was used for the abovementioned measurements.

### 3.9. Statistical Analysis

All the analyses were carried out in triplicate, through which means and standard deviations were calculated. In addition, one-way analysis of variance and Tukey’s test were carried out (Statistica 12.0, StatSoft, Krakow, Poland) to determine the significance of the differences (*α* = 0.05) between the means. Significantly different results were represented by different letters (a, b, c, …).

## 4. Conclusions

The results of this study showed that dry and wet micronization of the leaves and stems produced different results. Differences in appearance, coloration, particle size, the content of extractable phenolic compounds, TPC, and AA were observed between the powders. Wet micronization resulted in darker green powders, whereas dry micronization resulted in light green powders. The stem powders had a less intense green color than the leaf powders. Both the dry and wet micronization of the leaves and stems significantly decreased the dimensions of the particles; however, in dry micronization, the dimensions were significantly smaller. The dry-micronized leaf and stem powders had a Dv50 of 19.5 and 10.1 µm, whereas the wet-micronized leaf and stem powders had a Dv50 of 84.6 and 112.5 µm. More phenolic compounds, such as o-coumaric acid and gallic acid, were extracted from the dry-micronized powders. The 3-hydroxyphenylacetic acid content was also higher in the dry-micronized leaves compared with the wet-micronized ones. The lowest TPC and AA were observed in the wet-micronized leaf and stem powders. The dry micronization of the spinach stems significantly increased the TPC and AA of these powders. Thus, in addition to leaf powders, stem powders, when prepared appropriately, can be a valuable component of functional foods.

## Figures and Tables

**Table 1:** Results of the particle sizes of micronized spinach leaves and stems.

	D [3;2] (µm)	D [4;3] (µm)	Dv10 (µm)	Dv50 (µm)	Dv90 (µm)
CL	65.6 ± 3.27 ^e^	315.3 ± 70.22 ^e^	33.7 ± 1.2 ^e^	182.3 ± 10.69 ^e^	666.3 ± 26.31 ^e^
CS	74.5 ± 5.80 ^f^	313.0 ± 16.46 ^e^	41.5 ± 4.9 ^f^	210.7 ± 18.34 ^f^	743.7 ± 29.69 ^f^
WML	27.5 ± 3.02 ^c^	121.4 ± 13.35 ^c^	14.5 ± 1.4 ^c^	84.6 ± 16.79 ^c^	252.7 ± 11.64 ^c^
WMS	40.0 ± 5.24 ^d^	155.7 ± 10.79 ^d^	20.8 ± 2.1 ^d^	112.5 ± 13.85 ^d^	353.0 ± 3.61 ^d^
DML	6.4 ± 0.15 ^b^	28.1 ± 1.18 ^b^	3.7 ± 0.1 ^b^	19.5 ± 0.26 ^b^	62.1 ± 3.56 ^b^
DMS	3.3 ± 0.08 ^a^	16.7 ± 1.71 ^a^	1.6 ± 0.0 ^a^	10.1 ± 0.28 ^a^	40.8 ± 2.66 ^a^

D [3;2] -PO-weighted average particle dimensions of the sample surface area; D [4;3]- PO-weighted average particle dimensions of the sample volume; Dv10, Dv50, and Dv90-particle size below their value is 10, 50, and 90% of the total volume of the sample. CL-control freeze-dried spinach leaf powder without micronization, CS-control freeze-dried spinach stem powder without micronization, WML-wet-micronized freeze-dried spinach leaf powder, WMS-wet-micronized freeze-dried spinach stem powder, DML—dry-micronized freeze-dried spinach leaf powder, DMS-dry-micronized freeze-dried spinach stem powder. a–f-values in the same column marked with different letters are significantly (*α* = 0.05) different.

**Table 2:** Identification of phenolic compounds in micronized spinach leaves and stems.

Kind of Sample	3-Hydroxyphenylacetic Acid (mg/kg)	4-Hydroxyphenylacetic Acid (mg/kg)	o-Coumaric Acid (mg/kg)	*p*-Coumaric Acid (mg/kg)	Gallic Acid (mg/kg)	Ferulic Acid (mg/kg)	Caffeic Acid (mg/kg)
CL	2.08 ± 0.15 ^d^	0.99 ± 0.03 ^a^	8.01 ± 0.19 ^b^	3.05 ± 0.09 ^a^	12.32 ± 0.18 ^b^	40.33 ± 0.30 ^c^	33.97 ± 0.08 ^d^
CS	1.01 ± 0.12 ^a^	2.78 ± 0.10 ^b^	8.37 ± 0.14 ^c^	3.24 ± 0.11 ^b^	8.19 ± 0.13 ^a^	22.87 ± 0.22 ^a^	2.52 ± 0.21 ^b^
WML	4.69 ± 0.12 ^f^	3.79 ± 0.10 ^d^	7.82 ± 0.08 ^ab^	3.56 ± 0.12 ^c^	17.47 ± 0.21 ^d^	43.00 ± 0.09 ^d^	25.05 ± 0.28 ^c^
WMS	2.84 ± 0.09 ^e^	5.32 ± 0.13 ^e^	7.56 ± 0.21 ^a^	4.05 ± 0.10 ^d^	19.75 ± 0.31 ^e^	40.38 ± 0.13 ^c^	1.68 ± 0.11 ^a^
DML	1.49 ± 0.10 ^c^	1.15 ± 0.08 ^a^	8.71 ± 0.14 ^d^	3.16 ± 0.12 ^ab^	14.02 ± 0.16 ^c^	40.77 ± 0.31 ^c^	35.19 ± 0.13 ^e^
DMS	1.29 ± 0.06 ^b^	3.25 ± 0.11 ^c^	9.51 ± 0.10 ^e^	3.50 ± 0.13 ^c^	12.43 ± 0.25 ^b^	31.81 ± 0.37 ^b^	2.60 ± 0.05 ^b^

CL-control freeze-dried spinach leaf powder without micronization, CS-control freeze-dried spinach stem powder without micronization, WML-wet-micronized freeze-dried spinach leaf powder, WMS-wet-micronized freeze-dried spinach stem powder, DML-dry-micronized freeze-dried spinach leaf powder, DMS-dry-micronized freeze-dried spinach stem powder. a–f-values in the same column marked with different letters are significantly (*α* = 0.05) different.

**Table 3:** Total phenolic content and antioxidant activity of the spinach leaves and stems.

	TPC (mg GAE/g DM)	EC_50 DPPH_ (mg DM/mL)	EC_50 ABTS_ (mg DM/mL)
CL	0.38 ± 0.04 ^a^	273.79 ± 8.39 ^b^	333.48 ± 2.93 ^d^
CS	0.37 ± 0.03 ^ab^	759.25 ± 28.67 ^d^	528.53 ± 17.95 ^e^
WML	0.35 ± 0.02 ^ab^	384.79 ± 13.17 ^c^	254.92 ± 1.64 ^c^
WMS	0.30 ± 0.03 ^b^	898.45 ± 17.29 ^e^	888.30 ± 4.16 ^f^
DML	0.42 ± 0.02 ^a^	189.43 ± 0.72 ^a^	168.90 ± 3.02 ^b^
DMS	0.67 ± 0.04 ^c^	185.79 ± 2.43 ^a^	139.45 ± 4.13 ^a^

TPC-total phenolic content, DPPH-antioxidant activity against DPPH, ABTS-antioxidant activity against ABTS, EC50 value-the half-maximal inhibitory concentration, which was calculated by interpolating the dose–response curves and calculated as the concentration at which the tested extract showed 50% of the maximum inhibition based on the fitted model, DM-dry mass, CL-control freeze-dried spinach leaf powder without micronization, CS-control freeze-dried spinach stem powder without micronization, WML-wet-micronized freeze-dried spinach leaf powder, WMS-wet-micronized freeze-dried spinach stem powder, DML-dry-micronized freeze-dried spinach leaf powder, DMS-dry-micronized freeze-dried spinach stem powders. a–f-values in the same column marked with different letters are significantly (*α* = 0.05) different.

## Data Availability

Not applicable.

## Sample Availability

Samples of the compounds are not available from the authors.

## References

[1] Dhiman A., Prabhakar P.K. (2021). Micronization in food processing: A comprehensive review of mechanistic approach, physicochemical, functional properties and self-stability of micronized food materials. J. Food Eng..

[2] Dziki D., Tarasiuk W., Gawlik-Dziki U. (2021). Micronized oat husk: Particle size distribution, phenolic acid profile and antioxidant properties. Materials.

[3] Nemzer B., Al-Taher F., Abshiru N. (2021). Extraction and natural bioactive molecules characterization in spinach, kale and purslane: A comparative study. Molecules.

[4] Roberts J.L., Moreau R. (2016). Functional properties of spinach (*Spinacia oleracea* L.) phytochemicals and bioactives. Food Funct..

[5] Collins K., Zhao K., Jiao C., Xu C., Cai X., Wang X., Ge C., Dai S., Wang Q., Wang Q. (2019). SpinachBase: A central portal for spinach genomics. Database.

[6] Shukla P., Kumar R., Raib A.K. (2016). Detection of Minerals in Green Leafy Vegetables Using Laser Induced Breakdown Spectroscopy. J. Appl. Spectrosc..

[7] Qin J., Shi A., Mou B., Grusak M.A., Weng Y., Ravelombola W., Bhattarai G., Dong L., Yang W. (2017). Genetic diversity and association mapping of mineral element concentrations in spinach leaves. BMC Genom..

[8] Junejo S.A., Rashid A., Yang L., Xu Y., Kraithong S., Zhou Y. (2021). Effects of spinach powder on the physicochemical and antioxidant properties of durum wheat bread. LWT.

[9] El-Sayed S.M. (2020). Use of spinach powder as functional ingredient in the manufacture of UF-Soft cheese. Heliyon.

[10] Waseem M., Akhtar S., Manzoor M.F., Mirani A.A., Ali Z., Ismail T., Ahmad N., Karrar E. (2021). Nutritional characterization and food value addition properties of dehydrated spinach powder. Food Sci. Nutr..

[11] Doymaz I. (2009). Thin-layer drying of spinach leaves in a convective dryer. J. Food Process Eng..

[12] Vargas L., Kapoor R., Nemzer B., Feng H. (2022). Application of different drying methods for evaluation of phytochemical content and physical properties of broccoli, kale, and spinach. LWT.

[13] Çalışkan Koç G., Nur Dirim S. (2017). Spray Drying of Spinach Juice: Characterization, Chemical Composition, and Storage. J. Food Sci..

[14] Dadali G., Demirhan E., Özbek B. (2007). Color change kinetics of spinach undergoing microwave drying. Dry. Technol..

[15] Bajgai T.R., Hashinaga F. (2001). Drying of spinach with a high electric field. Dry. Technol..

[16] Song J., Zhang H.Y., Yuan J., Zeng C.Z., Mu Y.W., Kang S.J., Li Y.X., Gou L.N. (2021). Comparison of Physicochemical Properties of Spinach Powder Using Different Drying Methods. Mod. Food Sci. Technol..

[17] Ozkan I.A., Akbudak B., Akbudak N. (2007). Microwave drying characteristics of spinach. J. Food Eng..

[18] Yamakage K., Yamada T., Takahashi K., Takaki K., Komuro M., Sasaki K., Aoki H., Kamagata J., Koide S., Orikasa T. (2021). Impact of pre-treatment with pulsed electric field on drying rate and changes in spinach quality during hot air drying. Innov. Food Sci. Emerg. Technol..

[19] Watanabe T., Orikasa T., Shono H., Koide S., Ando Y., Shiina T., Tagawa A. (2016). The influence of inhibit avoid water defect responses by heat pretreatment on hot air drying rate of spinach. J. Food Eng..

[20] Sahin F.H., Acikgoz F.E., Eremkere M., Aktas T. (2019). Physical and mechanical properties and influence of drying techniques on drying characteristics and some quality parameters of malabar spinach (*Basella alba* L.). Fresenius Environ. Bull..

[21] Lefsrud M., Kopsell D., Sams C., Wills J., Both A.J. (2008). Dry matter content and stability of carotenoids in kale and spinach during drying. HortScience.

[22] Wang R., Wang T., Zheng Q., Hu X., Zhang Y., Liao X. (2012). Effects of high hydrostatic pressure on color of spinach purée and related properties. J. Sci. Food Agric..

[23] Manolopoulou E., Varzakas T. (2016). Effect of temperature in color changes of green vegetables. Curr. Res. Nutr. Food Sci..

[24] Bartos C., Szabó-Révész P., Bartos C., Katona G., Jójárt-Laczkovich O., Ambrus R. (2016). The Effect of an Optimized Wet Milling Technology on the Crystallinity, Morphology and Dissolution Properties of Micro- and Nanonized Meloxicam. Molecules.

[25] Bender A.B.B., Speroni C.S., Moro K.I.B., Morisso F.D.P., dos Santos D.R., da Silva L.P., Penna N.G. (2020). Effects of micronization on dietary fiber composition, physicochemical properties, phenolic compounds, and antioxidant capacity of grape pomace and its dietary fiber concentrate. LWT.

[26] Singh J., Jayaprakasha G.K., Patil B.S. (2018). Extraction, identification, and potential health benefits of spinach flavonoids: A review. Advances in Plant Phenolics: From Chemistry to Human Health.

[27] Speroni C.S., Bender A.B.B., Stiebe J., Ballus C.A., Ávila P.F., Goldbeck R., Morisso F.D.P., da Silva L.P., Emanuelli T. (2020). Granulometric fractionation and micronization: A process for increasing soluble dietary fiber content and improving technological and functional properties of olive pomace. LWT.

[28] Różyło R., Szymańska-Chargot M., Gawlik-Dziki U., Dziki D. (2021). Spectroscopic, mineral, and antioxidant characteristics of blue colored powders prepared from cornflower aqueous extracts. Food Chem..

[29] Sobaszek P., Różyło R., Dziki L., Gawlik-Dziki U., Biernacka B., Panasiewicz M. (2020). Evaluation of color, texture, sensory and antioxidant properties of gels composed of freeze-dried maqui berries and agave sugar. Processes.

[30] Ziemichód A., Różyło R., Dziki D. (2020). Impact of Whole and Ground-by-Knife and Ball Mill Flax Seeds on the Physical and Sensorial Properties of Gluten Free-Bread. Processes.

[31] Isidorov V.A., Smolewska M., Purzyńska-Pugacewicz A., Tyszkiewicz Z. (2010). Chemical composition of volatile and extractive compounds of pine and spruce leaf litter. Biogeosciences.

[32] Różyło R., Piekut J., Wójcik M., Kozłowicz K., Smolewska M., Krajewska M., Szmigielski M., Bourekoua H. (2021). Black cumin pressing waste material as a functional additive for starch bread. Materials.

[33] Singleton V.L., Rossi J. (1965). Colorimetry of Total Phenolics With Phosphomolybdic. Am. J. Enol. Vitic..

[34] Brand-Williams W., Cuvelier M.E., Berset C. (1995). Use of a free radical method to evaluate antioxidant activity. LWT-Food Sci. Technol..

[35] Re R., Pellegrini N., Proteggente A., Pannala A., Yang M., Rice-Evans C. (1999). Antioxidant activity applying an improved ABTS radical cation decolorization assay. Free Radic. Biol. Med..

